# Factors Associated With Normal Flow (TIMI 3) After Thrombolysis With Streptokinase in ST-Elevation Myocardial Infarction: A Prospective Observational Study

**DOI:** 10.7759/cureus.12758

**Published:** 2021-01-18

**Authors:** Karthik Raghuram, Surendran Deepanjali, Ajith Ananthakrishna Pillai

**Affiliations:** 1 Medicine, Jawaharlal Institute of Postgraduate Medical Education and Research, Puducherry, IND; 2 Cardiology, Jawaharlal Institute of Postgraduate Medical Education and Research, Puducherry, IND

**Keywords:** timi 3 flow, st-elevation myocardial infarction, infarct-related artery

## Abstract

Background

Thrombolysis with streptokinase (STK) is the most widely used reperfusion strategy for ST elevation myocardial infarction (STEMI) in India. Achieving full reperfusion as evidenced by thrombolysis in myocardial infarction (TIMI) flow grade 3 in coronary angiography (CAG) is associated with better outcomes. Recent studies show that hematological indices like neutrophil-lymphocyte ratio (NLR) and mean platelet volume (MPV) estimated before thrombolysis could predict TIMI 3 flow. We studied clinical, electrocardiographic and hematological parameters associated with TIMI 3 flow after thrombolysis with STK.

Methods

We prospectively studied 201 adult patients with STEMI presenting within 12 hours of onset of chest pain. Before thrombolysis, blood sample was collected for estimating NLR and MPV. Timing of CAG after thrombolysis was decided by consultant cardiologists. Patients were followed up for one month after discharge.

Results

Of 201 patients, 162 (81%) had relief of chest pain and 131 (65%) had ST segment recovery of ≥50% at 90 minutes after thrombolysis. CAG was performed within median (IQR) of four (3-5) days after thrombolysis. TIMI 3 flow was observed in 112 (56%) patients. NLR and MPV had no significant association with TIMI 3 flow. In multivariable analysis, ST-segment recovery of ≥50% at 90 minutes was associated with TIMI 3 flow (adjusted OR 3.47, 95% CI: 1.84-6.53, P= <0.001). Of 198 patients followed up for one month after discharge, 13 (6.5%) died.

Conclusions

In patients with STEMI, ST-segment recovery of ≥50% at 90 minutes after thrombolysis with STK predicted TIMI 3 flow independently. NLR and MPV values were not predictive of TIMI 3 flow.

## Introduction

Primary percutaneous coronary intervention (PPCI) is accepted as the most preferred reperfusion strategy for ST elevation myocardial infarction (STEMI). However, in many low and middle income countries (LMICs) like India, thrombolysis with streptokinase (STK) remains the most commonly used reperfusion strategy [1]. The primary aim of thrombolysis is to achieve rapid and complete patency of the infarct-related artery (IRA). Existing evidence suggests that full reperfusion as designated by thrombolysis in myocardial infarction (TIMI) flow grade 3 in coronary angiography (CAG) is associated with better myocardial salvage and decreased mortality [2]. Previous studies show that factors such as type of fibrinolytic agent and absence of T wave inversion in baseline electrocardiogram (ECG) are associated with achieving TIMI 3 flow after thrombolysis [3,4]. Hematological parameters of inflammation such as neutrophil-lymphocyte ratio (NLR) and mean platelet volume (MPV) were also found to be predictive of TIMI 3 flow after thrombolysis [5,6]. We studied the clinical, electrocardiographic and hematological parameters which were associated with finding angiographic TIMI 3 flow in patients presenting with STEMI who underwent thrombolysis with STK at our center. We also studied the one-month mortality of these patients.

## Materials and methods

We conducted a prospective observational study in the emergency and medical wards of Jawaharlal Institute of Postgraduate Medical Education and Research, a tertiary care hospital in Puducherry, Southern India. The study protocol was approved by the Institute Ethics Committee (JIP/IEC/2015/17/665). Between October 2015 and June 2017, we studied adult patients presenting within 12 hours of onset of chest pain to the emergency department who met the electrocardiographic (ECG) criteria for STEMI and underwent thrombolysis with STK. We excluded patients who underwent thrombolysis elsewhere and those who did not undergo CAG after thrombolysis. All patients received intravenous STK 1.5 million units administered over 60 minutes. An ethylenediaminetetraacetic acid (EDTA) sample for NLR and MPV was collected before administering STK. The samples were processed using an automated haematology analyser (Sysmex XT-1800i, Sysmex Corp., Kobe, Japan).

Following thrombolysis, ECGs were repeated at 90 and 120 minutes to observe ST segment recovery. Patients received the usual standard of care medications such as subcutaneous heparin, antiplatelet agents, statins, beta-blockers and ACE inhibitors. Timing of CAG after thrombolysis was decided by consultant cardiologists which varied depending on availability of cardiac catheterization laboratory. The TIMI flow grades are TIMI 0: absence of any antegrade flow beyond a coronary occlusion; TIMI 1: faint antegrade coronary flow beyond the occlusion, although filling of the distal coronary bed is incomplete; TIMI 2: delayed or sluggish antegrade flow with complete filling of the distal territory and TIMI 3: normal flow that fills the distal coronary bed completely [7]. Patients or their attendants were contacted telephonically one month later to determine patients’ survival status and to collect information on re-admissions or persistent symptoms.

Statistical analysis

To find out the factors associated with TIMI 3 flow we performed chi-square test for categorical variables and independent sample t-test or Wilcoxon rank-sum test for continuous variables. Those variables found to have a P-value of <0.1 were entered into a multivariable logistic regression model to find out factors independently associated with TIMI 3 flow. A two-sided P-value <0.05 was considered significant. All analyses were performed using the statistical software package Stata/IC 12.1 for Windows (StataCorp LP, College Station, Texas, USA).

## Results

**Figure 1:**
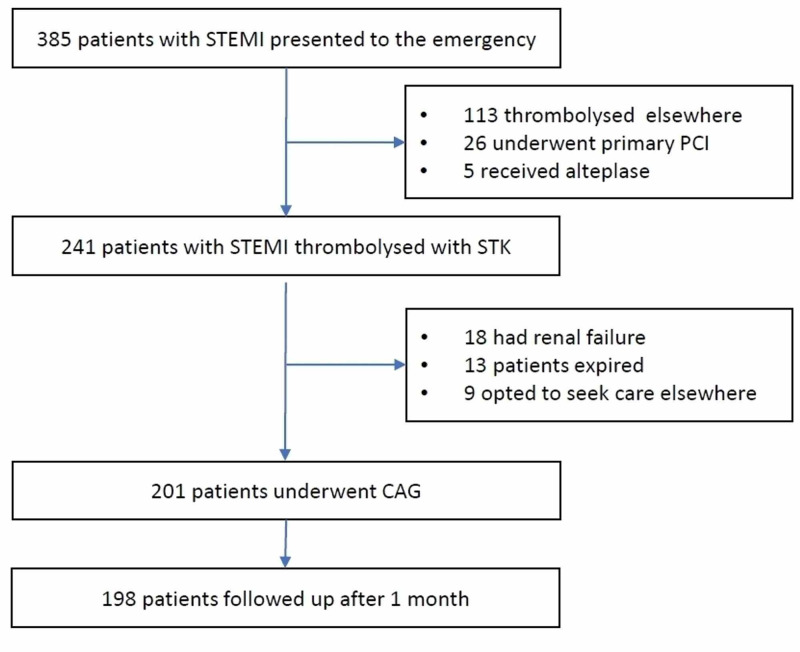
The STROBE flow diagram STROBE: Strengthening the reporting of observational studies in epidemiology; STEMI: ST elevation myocardial infarction; PCI: Percutaneous coronary intervention; STK: Streptokinase; CAG: Coronary angiography.

We included 201 patients. The STROBE (Strengthening the Reporting of Observational Studies in Epidemiology) diagram (Figure 1) depicts the inclusion of participants in the study. The median (IQR) age of the patients was 55 (45-61) years, majority being males (168, 84%). Of the 201 patients, 180 (90%) were in Killip class 1, 16 (7.9%) in class 2, five (2.5) in class 3 while no patient was in class 4 at presentation. According to the baseline ECG, 111 (55.2%) had anteroseptal wall involvement and 90 (44.7%) had inferior wall involvement. At 90 minutes after thrombolysis, 162 (81%) had relief of chest pain and 131 (65%) had ST segment recovery of at least 50% in the ECG. At 120 minutes, further 26 patients reported relief of chest pain of whom 15 had resolution of ST segment elevation in the ECG. CAG was done within a median (IQR) of 4 (3-5) days after thrombolysis. TIMI 3 flow in the infarct-related artery was observed in 112 (56%) patients. The clinical, ECG and laboratory characteristics of patients with and without TIMI 3 flow is given in Table 1.

**Table 1:** Comparison of clinical, ECG and laboratory features in patients with TIMI 3 flow in CAG to those with TIMI 0, 1 or 2 flow ECG: Electrocardiogram; TIMI: Thrombolysis in myocardial infarction; CAG: Coronary angiography; SD: Standard deviation; IQR: Interquartile range.

Characteristics	Patients with TIMI 3 flow, N = 112	Patients with TIMI 0, 1 or 2, N = 89	P-value
Male sex, n (%)	95 (85)	73 (82)	0.595
Age (years), mean (±SD)	53 (±11)	55 (±11)	0.129
Hypertension	32 (29)	27 (30)	0.785
Diabetes, n (%)	34 (30)	32 (36)	0.401
Smoking, n (%)	53 (47)	41 (46)	0.860
Body mass index, kg/m^2^, mean (±SD)	24.4 (±2.1)	24.5 (±2.1)	0.775
Systolic blood pressure at admission, mm of Hg, mean (±SD)	126 (±21)	121 (±23)	0.105
Diastolic blood pressure at admission, mm of Hg, mean (±SD)	78.4 (±13)	76.1 (±12)	0.202
Duration of chest pain (hours), mean (±SD)	4.4 (±2.4)	5.1 (±2.5)	0.039
Door to needle time in minutes, mean (±SD)	38 (±22)	38 (±23)	0.946
Total ischemic time in hours, mean (±SD)	4.9 (±2.5)	5.7 (±2.6)	0.030
Q waves in baseline ECG, n (%)	70 (63)	61 (69)	0.372
Inverted T waves in the ST elevated leads in baseline ECG, n (%)	26 (23)	36 (40)	0.009
Fragmented QRS complexes in baseline ECG, n (%)	94 (84)	68 (76)	0.180
Reduction of chest pain at 90 minutes, n (%)	111 (99)	61 (69)	<0.001
ST-segment elevation recovery by ≥50% at 90 minutes, n (%)	88 (79)	43 (48)	<0.001
Reduction of chest pain at 120 minutes, n (%)	10 (9)	16 (18)	0.044
ST-segment elevation recovery by ≥50% at 120 minutes, n (%)	7 (6)	8 (9)	0.254
Time gap between thrombolysis and CAG in days, median (IQR)	4 (3-5)	4 (3-5)	0.425
Neutrophil-lymphocyte ratio, median (IQR)	4.6 (3.04-7.5)	5.06 (3.5-7.2)	0.239
Mean platelet volume, femtolitre, mean (±SD)	10 (±0.66)	10 (±0.75)	0.952

Multivariable logistic regression analysis

We performed multivariable logistic regression analysis of variables which were significant in the univariate analysis. However, to avoid multi-collinearity, we omitted two variables; duration of chest pain and resolution of chest pain at 120 minutes. We also found that resolution of chest pain at 90 minutes was significantly associated with ST-segment recovery at 90 minutes (odds ratio 154.37, 95% CI: 20.42-1167; P < 0.001). Since the ECG parameter is more objective, we omitted chest pain relief at 90 minutes from the model. The final model had three variables of which ST-segment recovery of ≥50% at 90 minutes was found to be independently associated with TIMI 3 flow (Table 2). However, McFadden’s Pseudo R2 for this model was 0.089 which indicates that just less than 10% of variability of parameters is explained by the model [8].

**Table 2:** Multivariable logistic regression analysis of factors associated with TIMI 3 flow in univariate analysis TIMI: Thrombolysis in myocardial infarction

Variables	Adjusted odds ratio (95% CI)	P-value
Total ischemic time	0.96 (0.84-1.08)	0.464
Presence of inverted T waves in the ST elevated leads in the baseline ECG	0.55 (0.29-1.06)	0.075
ST-segment elevation recovery by ≥50% at 90 minutes	3.47 (1.84-6.53)	<0.001

Mortality at one month

Of 198 patients who were followed up for one month after discharge, 13 (6.5%) died. There were nine males and four females. Of the 13, three had undergone percutaneous intervention with stent placement after discharge. There was no statistically significant association with lower TIMI flow grades and mortality (OR [95% CI] 0.36 [0.11-1.2]); P = 0.084. However, our study was not adequately powered to show difference in mortality rates.

## Discussion

We observed that in a population of 201 patients with STEMI treated with STK, ST-segment recovery of >50% at 90 minutes after thrombolysis independently predicts TIMI 3 flow. Although the lower total ischemic time, absence of T wave inversions in the baseline ECG and resolution of chest pain at 90 minutes were also significantly associated with patent IRA in univariate analysis, they were not significantly predictive in the multivariable model. We also observed a one-month mortality after discharge of 6.5%.

The proportion of patients with TIMI 3 flow (56%) as found in this study is similar to a full angiographic patency of 51% at 5-7 days which was observed in the STK with subcutaneous heparin arm of the GUSTO trial [9]. In GUSTO Angiographic Study, investigators studied the clinical predictors of early (90 minutes after thrombolysis) IRA patency [3]. They found that use of an accelerated tissue plasminogen activator (t-PA) regimen, anatomical localization of IRA [right coronary artery (RCA)/left circumflex artery (LCx) vs. left anterior descending (LAD)], body weight and smoking were predictive factors. However, we found no association of a particular IRA, BMI or smoking status with TIMI 3 flow. This could possibly happen because of the smaller sample size of our study. However, it is notable that other similarly large data sets like the pooled PAMI trial data found that particular localization of infarct vessel or current smoking did not predict TIMI3 flow [10].

A study similar to ours, which assessed predictors of coronary artery patency after thrombolysis with STK, was carried out by Gokhroo et al. in Rajasthan [11]. They included 100 patients who underwent CAG within 48 hours of thrombolysis with STK. They found that a patent IRA (defined as TIMI 2 or 3 flow) was present in 76% of patients. Of note, they observed that patency was more in patients presenting early (0-2 hours of chest pain onset). Indeed, the impact of total ischemic time on IRA patency has been observed in other studies also [12]. Although, we did not observe any significant association of total ischemic time with TIMI 3 flow, it is quite intuitive to minimize pre-hospital delays for achieving optimal efficacy of any reperfusion strategy.

We found that reduction in ST-segment elevation by >50% in the 90-minute ECG was associated with finding full angiographic patency, a finding which has been previously reported [13]. Studies show that ST-segment recovery also has prognostic significance in terms of predicting mortality and development of heart failure [14]. Conversely, patients without ST-segment recovery with persistent chest pain could benefit more by a pharmaco-invasive strategy. We found no significant association of NLR and MPV values and TIMI 3 flow. Some studies indicated that increased NLR and MPV could predict failed thrombolysis, no-reflow phenomenon in PCI and higher mortality [15,16]. However, these indices are non-specific markers of inflammation and have been observed to be elevated in non-cardiac diseases also [17,18].

The major limitation of our study is that we did not collect echocardiographic data to study the association of left ventricular function and IRA patency. Also, our follow-up period of one month was relatively short. The regression model evolved from our study data was modest in accounting for the variability in the outcome. However, this was not totally unexpected since multiple potential factors are known to influence TIMI flow grades [19,20]. Future studies from India evaluating the outcomes of thrombolysis with STK in STEMI should study incidence of major adverse cardiac events and mortality.

## Conclusions

This study examined the clinical, electrocardiographic and hematological parameters associated with TIMI 3 flow after thrombolysis with STK in patients who presented to the emergency department with STEMI. We observed that among 201 patients with STEMI, around 60% had TIMI 3 flow following thrombolysis. In the univariate analysis, duration of chest pain, total ischemic time, inverted T waves in baseline ECG, reduction of chest pain at 90 and 120 minutes, and ST-segment recovery ≥50% in ECG at 90 minutes were found to be associated with TIMI 3 flow. However, age, sex and history of smoking were not found to have any significant association with TIMI 3 flow. NLR and MPV values were also not predictive of the same. Multivariable analysis found that ST-segment recovery ≥50% in ECG at 90 minutes was independently associated with TIMI 3 flow. The observed one-month mortality was 6.5% in patients who were followed up.
